# Ultrasonic extraction and antioxidant evaluation of oat saponins

**DOI:** 10.1016/j.ultsonch.2024.106989

**Published:** 2024-07-18

**Authors:** Lina Zhang, Jianing Li, Yingrui Huo, Wenping Yang, Jie Chen, Zhiqiang Gao, Zhenping Yang

**Affiliations:** a College of Agronomy, Shanxi Agricultural University, Taigu 030800, PR China; b Collaborative Innovation Center for High-quality and Efficient Production of Characteristic Crops on the Loess Plateau, Jointly Built by the Province and the Ministry, Taigu 030800, PR China; c College of Life Sciences, North China University of Science and Technology, Tangshan 063210, PR China

**Keywords:** Oat Saponins, Ultrasound-assisted extraction, Response surface optimization, Antioxidation

## Abstract

The aim of this study is to optimize the extraction process of oat saponins (Os) and to evaluate their antioxidant potential. Single factor experiment, response surface optimization design, and orthogonal test were employed to optimize the process of ultrasonic-assisted extraction of Os, and the optimal extraction conditions were as followed: ethanol volume fraction of 80 %, material-solvent ratio of 1:14, ultrasonic power of 400 W, ultrasonic time of 25 min, extraction temperature of 60℃, extraction time of 180 min, and the extraction rate of Os was 0.317 %±0.105 %. Using the method, the crude extract of Os was prepared and its abilities of scavenging radicals in vitro and inhibiting protein oxidation in pork were determined, with ascorbic acid (Vc) as the control. Results revealed that the scavenging ability of Os against DPPH radical, hydroxyl radical (·OH) and superoxide anion (O_2_^–^) increased with the concentration of Os. Interestingly, the scavenging abilities of Os against DPPH and O_2_^–^ were far lower than that of Vc, but its scavenging ability against ·OH was very close to that of Vc, reaching 84.59 % and 96.33 %, respectively. Furthermore, the experiments of pork storage and Fenton oxidation system showed that Os with 0.09–0.72 mg/mL could reduce the production of carbonyl (8.49 %-50.05 %) and the oxidation of total sulfhydryl (1.29 %-25.86 %), and effectively inhibit the oxidation of protein in pork by 7.82 %–22.53 %. The results of this study will provide a theoretical basis for the application of oat saponins as a natural anti-protein oxidant in meat processing and storage.

## Highlights

• Among the cereals we consume daily, only oats contain saponins.
• The ultrasonic-assisted extraction process of oat saponins was optimized.
• The antioxidant effects of oat saponins as natural peroxidants were evaluated, including antioxidant activity in vitro and anti-protein oxidation ability during pork storage. The results showed that the DPPH, ·OH and O^2–^ free radical activities reduced with increasing concentration of the Os. In addition, Os could effectively inhibit the oxidation of protein.

## Graphical abstract

**Figure:**
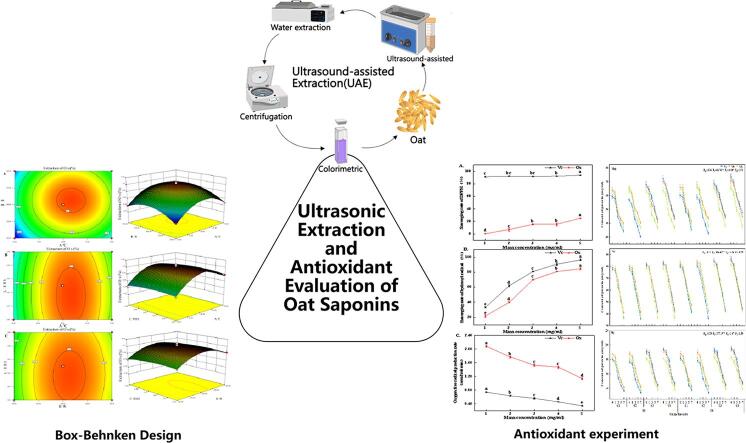

## 1 Introduction

In the daily diet, as the main source of protein, meat and its products occupy an important position. However, during the process and storage of meat and its products, their proteins are often vulnerable to damage by free radicals or reactive oxygen, resulting in oxidative deterioration, which further affects the functional properties and digestibility of proteins, and ultimately damages their quality characteristics [1], [2]. At present, synthetic antioxidants, such as butylhydroxyanisole (BHA), propyl gallate (PG), *tert*-butylhydroquinone (TBHQ) and dibutylhydroxytoluene (BHT), are widely used in the meat industry to combat the oxidation of fats and proteins [3], [4]. However, given the potential toxicological risks of these synthetic antioxidants, the search for natural antioxidants as an alternative has become an inevitable trend in industry.

Oat saponins (Os) are a type of natural compounds with multiple biological activities, such as excellent anti-diabetes [5], prevention of bone diseases [6], protection of the stomach [7], anti-cancer [8] and other effects, and also are a kind of natural foods and drug homologous substances. The saponins in oat grains and aboveground by-products (stems, brans, etc.) are mainly Avenacoside-A and Avenacoside-B. Based on the basic carbon chain structure, these saponins also contain the trisugar unit at C-3 and the glucose unit at C-26, which are crucial in the resistance to microorganisms [9]. Some scholars studied the effects of oat saponins on plasma and liver lipids of mice, and their research results showed that cholesterol content in liver was significantly reduced, and oat saponins had anti-lipid oxidation effects [10]. When Kim reported the influence of diet control on inflammation, he found that oat saponins had a good protective effect on inflammation caused by cellular oxidative damage [11]. Although the anti-lipid oxidation effects of oat saponins have been extensively studied, their antioxidant effects on proteins in meat storage or induced by Fenton oxidation system (Fe^3+^, ascorbic acid (Vc), and different concentrations of H_2_O_2_) have been less reported.

To find a fast and effective method for extraction and determination of oat saponins is an important prerequisite for making full use of oat saponins. Most researchers used high performance liquid chromatography-mass spectrometry to analyze the content of saponins in oat leaves and bran [12], [13], [14], and the average content ranged from 0.001 % to 0.090 %. This method can be used for qualitative and quantitative analysis of saponins, but which requires certain professional knowledge and skills, high operational complexity, and strict requirements for test environment. Vanillal-acetic acid-perchloric acid color development method is widely used in histochemical localization, and saponins can produce color reactions from lavender to purple, so the spectrophotometric method is often used for the determination of saponins content [15], [16]. Some scholars used it to study the extraction process of oat saponins (in bran), and found that extraction solvent, extraction temperature, extraction time and solid–liquid ratio had a great impact on the extraction rate of oat saponins [17], [18]. However, due to the different control substances used in the study, there are some differences between the results of previous studies. In addition, both the traditional extraction method and cable extraction method require a lot of time, while ultrasonic assisted extraction has the advantages of fast, high efficiency and energy saving, etc. Some studies have shown that ultrasonic assisted extraction technology can greatly improve the extraction efficiency of natural compounds, and is a new generation of environmentally friendly, economic and feasible green extraction method [19], [20], [21], [22].

The aim of this study was to optimize the ultrasonic assisted extraction technique of oat saponins, and to prepare the crude extract of oat saponins using this method, and further to evaluate antioxidant properties of the extracts, including of the scavenging abilities of free radicals (DPPH, hydroxyl radical ·OH and superoxide anion O_2_^–^) in vitro, anti-protein oxidation ability during pork storage (in room temperature and cold storage), and inhibitory ability on the oxidation of myofibrin induced by Fenton oxidation system.

## 2 Materials and methods

### 2.1 Materials

Oat grains were obtained from High Latitude Crops Institute, Shanxi Academy, Shanxi Agricultural University, and they were processed by a high-speed tissue homogenizer (Tissuelyser-Ⅱ, QIAGEN, Germany) and stored in refrigerator until needed for experiment. The diosgenin used in this study was a standard product purchased from Sigma Company. The scavenging abilities of Os against free radicals (DPPH,·OH and O_2_^–^) were measured by using kits provided by Shanghai MLBIO Biotechnology Co., Ltd.

Fresh pork (Jinfen white pig, porcine M. longissimus dorsi muscle, 103–110 kg, 72 h after death, water content: 72.48 %, fat content: 5.11 %; AOAC 2000) was purchased from Shuanghui Specialty Store (Taigu, Shanxi, China). Excess fat and connective tissue were cut off, and the meat was stored at −20℃ until needed for experiment.

All chemicals and solvents were analytical grade, unless otherwise stated.

### 2.2 Methods

#### 2.2.1 Optimization of extraction technique of oat saponins

##### 2.2.1.1 Standard solution prepared

The dioscin standard solution: the dioscin of 10 mg was weighed accurately, dissolved in methanol, and transfered to a 10 mL volumetric flask.

The 5 % vanillin-glacial acetic acid solution: the vanillin of 0.5000 g was weighed accurately, dissolved in glacial acetic acid, and adjusted the volume in a 10 mL volumetric flask.

##### 2.2.1.2 Standard curve drawed

The standard solution mentioned above was accurately taken 10, 20, 30, 40, and 50 μL, and respectively dispensed into stoppered test tubes with 10 mL, then s upplemented the solution to 1 mL with methanol.The freshly prepared solution containing 5 % Vanillin-glacial acetic acid (0.2 mL) and perchloric acid (0.8 mL) were combined in these test tubes, mixed thoroughly and placed in a water bath at 60 °C for 15 min, and then cooled by immersing in ice water for a period of 5 min. In final, each test tube received an addition of glacial acetic acid (5 mL), shaken well and left to stand for 10 min. The absorbance was measured at 532 nm. The standard curve was drawn as below: y = 0.0015x + 0.0293 (R=0.9993) (Figure S1, where x is the mass concentration (mg/mL) values, y is the absorbance values). The extraction rate of Os was calculated using the following formula: Extraction rate of Os (%) = CV/M (C: the mass concentration of Os (mg/mL), V: the dilution factor, M: Sample of non-fat oats).

##### 2.2.1.3 Extraction process

The defatted oat grain flour was weighed 3.0 g, and dissolved in an ethanol solution and underwent ultrasound-assisted extraction. This extraction step was repeated three times. The extract was centrifuged at a speed of 3000 r/min for 10 min. A 1 mL aliquot was taken from the supernatant and dried in a water bath at 70 °C, then added equal volumes (2 mL each) of water and n-butanol saturated solution and ultrasonically mixed for 2 min. Finally, the mixture was centrifuged again at a speed of 3000 r/min for 30 min and taken 1 mL aliquot from its supernatant to measure its absorbance.

##### 2.2.1.4 Single-factor experimental design

The six factors, including of ethanol volume fraction, material-solvent ratio, ultrasonic power, ultrasonic time, extraction temperature, and extraction time, were set up to investigate their influences on the extraction rate of Os. In each single-factor experiment, one specific factor was varied while keeping the others constant. The optimal parameters determined in the previous single-factor experiment were utilized as the basis for each subsequent single-factor experiment. Each single-factor experiment was conducted in triplicate (Table S2).

##### 2.2.1.5 Orthogonal experiment

Based on the results of the single-factor experiments, the three main factors were identified and determined the appropriate ranges for extraction temperature, material-solvent ratio, and ultrasonic power. These factors were further validated using a three-factor, three-level regression orthogonal extraction process (Table S3).

##### 2.2.1.6 Response surface optimization design

Based on the results of the single-factor experiments, a response surface optimization experiment was conducted to consider three independent variables: extraction temperature (A), ultrasonic power (B), and material-solvent ratio (C), with three levels each, including of extraction temperatures of 50, 60 and 70℃, ultrasonic power of 300, 400 and 500 W, and material-solvent ratios of 1:12, 1:14 and 1:16, respectively. The three values of these variables were coded as −1, 0, and 1 respectively, representing low, medium, and high levels based on the Os extraction rate response value. Using the Design Expert 8.0.6 software with the Box-Behnken center combination design principle (BBD) to determine the optimal extraction conditions. The BBD method consisted of a total of seventeen experiments including twelve factorial points and five axial points. Table S4 provided specific values for each factor at different levels.

#### 2.2.2 Evaluation of antioxidant ability of oat saponins

##### 2.2.2.1 Antioxidant activity in vitro

The extract was collected and the ethanol was evaporated. The n-butanol was added three times for extraction, and the upper extract was obtained. After removing n-butanol from the upper extract, a crude extract of oat grain saponins sample was obtained. Its scavenging abilities to DPPH free radicals and hydroxyl radicals (·OH), as well as the inhibiting ability to superoxide anion (O_2_^–^) generating, were determined using the provided kit method. Five concentrations of Os were set for testing: 1 = 0.04 mg/mL, 2 = 0.09 mg/mL(C1), 3 = 0.18 mg/mL(C2), 4 = 0.36 mg/mL(C3), 5 = 0.72 mg/mL(C4). The L-ascorbic acid (Vc) with the five concentrations above mentioned and sterile water (CK) were set as the positive and negative controls, respectively. The results of the oxidation evaluation in vitro revealed that the antioxidant activity at 1 = 0.04 mg/mL did not meet the desired criteria, consequently, the concentration of 1 = 0.04 mg/mL was excluded from further consideration for meat protein antioxidant testing.

##### 2.2.2.2 Anti-protein oxidation ability during pork storage

**Raw materials preparation.** The pre-treated LD muscle, stored at −20℃, was transferred to a refrigerator set at 4℃ for 16 h. Subsequently, it was minced using a meat mincer (JRD07, SUPOR, China). The ground meat was divided into three equal portions, Each portion (50 g) was blended with 2 mL of Os, Vc and CK, respectively, Both Os and Vc had four concentrations of C1（0.09 mg/mL）, C2（0.18 mg/mL）, C3（0.36 mg/mL） and C4（0.72 mg/mL）, respectively. The prepared raw materials were sealed and stored at room temperature (RS:23℃) and refrigeration temperature(CS:4℃), respectively. On the days of 0, 1, 3, 5, and 7 during the storage period, these samples were taken to extract Mixed Muscle Proteins (Mep), Sarcoplasmic proteins (Sp),and Myofibrillar proteins (Mp) for analyzing the changes of sulfhydryls, carbonyls, and protein.

**Extraction of Mep, Sp and Mp.** According to Yang [23], the Mep, Sp, and Mp of the stored muscle sample were extracted. For Mep, 10 g of a muscle sample was weighed, mixed with 40 mL of cold deionized water and blended for 30 s at a speed of 13,000 r/min using an FSH-2 (A) High-speed dispersion homogenizer (RunHua, China). For Sp, 20 g of a muscle sample was mixed with 30 mL of cold deionized water and blended at a speed of 13,000 r/min for 30 s. Then the homogenate was centrifuged at 2000 g and 4 °C for 15 min, filtered through Whatman No.7 filter paper, and the supernatant (Sp fraction) was collected. Mp was isolated from separate muscle samples using a rigor buffer containing KCl (0.1 M), MgCl_2_ (2 mM), EGTA (1 mM), Na_2_HPO_4_(10 mM, pH7.0) [24]. The protein concentrations of Mep, Sp, and Mp were determined using the Biuret method [25].

**Total sulfhydryl group content.** The sulfhydryl content was determined according to Ellman [26] using 5,5′-dithio-bis(2-nitrobenzoic acid) (DTNB).

**Carbonyl content.** Carbonyl content was determined according to Oliver [27] and Lampila [28] using 2,4-dinitrophenylhy −drazine (DNPH) as the chromophore reactant.

##### 2.2.2.3 Anti-protein oxidation ability in Fenton oxidation system

According to Oliver's method [27], myofibrin (Mp) oxidation induced by the Fenton oxidation system was employed. The hydroxyl free radical, which is the most crucial and effective reactive oxygen species causing oxidative damage to meat proteins, can be generated through the Fenton reaction [29] catalyzed by iron ions in combination with hydrogen peroxide (H_2_O_2_) and cell reducing agents. Extensive research had been conducted on protein oxidation induced by Fenton oxidation systems (iron trioxide, ascorbic acid, and varying concentrations of H_2_O_2_) in the food system [30], [31]. In this study, ferric chloride and ascorbic acid were maintained at a concentration of 0.1 mM, while H_2_O_2_ concentrations ranged from 0 to 5 mM (0, 0.05, 0.1, 0.2, 0.5, 1 and 5 mM). Different concentrations of oat saponins solution were added to evaluate the extent of protein oxidation.

### 2.3 Data analysis

Data sorting was performed using Microsoft Excel 2010, and statistical analysis was conducted using SPSS 26.0. Statistical significance comparison was carried out using the ANOVA process and Duncan's method, and differences between treatments were displayed when *P*≤0.05. Response surface optimization prediction was performed using Design Expert 8.0.6, and figure plotting was conducted using Origin 2021.

## 3 Results

### 3.1 Optimization of extraction technique of oat saponins

#### 3.1.1 Single-factor experiment results

**Fig. 1:**
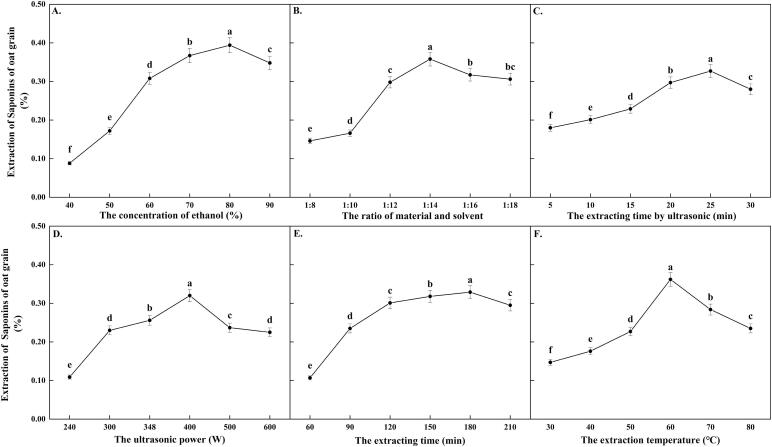
Effect of single-factor experiment for extraction of Saponins of oat grain (Os). A. The effect of different ethanol volume fraction for extraction of Os. B. The influence of different solid–liquid ratio for extraction of Os. C. The influence of different ultrasonic time for extraction of Os. D. Theeffect of different ultrasonic power for extraction of Os. E. Theinfluence of different extraction time for extraction of Os. F. Theinfluence of different extraction temperature for extraction of Os. Different letters indicated statistically significant differences between different treatments (LSD, *P* ≤ 0.05).

As seen in Fig. 1(A-F), all of the six tested single-factors significantly affected the extraction rate of Os and showed a trend of increasing first and then decreasing. Among all treatments, the extraction rate of Os had the highest value (0.384 ± 0.019 %) when the concentration of extracting solvent (the ethanol volume fraction) reached 80 %, and then decreased slightly when it exceeded 80 % (Fig. 1A).

Similarly, when the material-solvent ratio changed from 1:8 to 1:14, the Os extraction rate increased from 0.146 %±0.030 % to 0.358 %±0.017 %, and then decreased to 0.306 %±0.031 % with the ratio reaching 1:18, which indicated that the Os extraction rate became relatively stable from 1:14 to 1:18 (Fig. 1B).

As the ultrasonic time extended to 25 min, the Os extraction rate reached its highest value (0.327 %±0.020 %). But further extending the ultrasonic time, the decrease of Os extraction rate was not significant (Fig. 1C).

The extraction rate of Os reached its highest value (0.320 %±0.024 %) when the ultrasonic power was 400 W. The change amplitudes of the extraction rate were large when it was more than or less than 400 W (Fig. 1D).

The extraction time was extended from 60 min to 210 min, resulting in an increase in the extraction rate of Os from 0.107 % ± 0.032 % to 0.329 % ± 0.018 %. However, there were no significant changes observed at both ends of the highest value (Fig. 1E).

Finally, the highest extraction rate of Os(0.362 % ± 0.012 %) was achieved at 60 °C of the extraction temperature, and there were significant changes observed on both sides of the highest value(Fig. 1F).

Therefore, the material-solvent ratio (1:14), extraction temperature (60 °C), and ultrasonic poewr (400 W) were chosen as the optimal extraction factors for the subsequent orthogonal experiment.

#### 3.1.2 Orthogonal experiment results

We conducted a three-factor, three-level regression orthogonal experiment. The results showed that the extraction rate of Os ranged from 0.205 %±0.040 % to 0.303 %±0.028 %. According to the *P* value, the influence of each factor on the extraction rate of Os followed the order: A>B>C. It showed that the extraction temperature had a greater impact than ultrasonic power, which in turn had a greater impact than material-solvent ratio (Table 1). The results of the orthogonal inter-subject effect test indicated that the model was effective, with a *P* value of 0.011 and R^2^ of 0.989. Various factors significantly affected the extraction rate of Os, and there was a remarkable interaction effect between extraction temperature and ultrasonic power.

**Table 1:** Orthogonal interagent effect test.

SV	SS	df	MS	*F* value	*P* value	Partial Eta squared	Non-centrality parameter
Model	2.695a	32	0.084	93.573	0.011	0.999	2994.349
A	1.439	4	0.36	399.596	0.002	0.999	1598.383
B	1.129	4	0.282	313.747	0.003	0.998	1254.988
C	0.649	4	0.162	180.257	0.006	0.997	721.028
AB	0.113	4	0.028	31.318	0.031	0.984	125.272
AC	0.007	4	0.002	1.84	0.382	0.786	7.361
BC	0.003	4	0.001	0.926	0.578	0.649	3.703
ABC	0.017	8	0.002	2.385	0.329	0.905	19.078
Error	0.002	2	0.001				
Cor Total	229.011	35					
Cor Total（Adj）	2.697	34					
R^2^ = 0.999（ R^2^（Adj） = 0.989）							

#### 3.1.3 Response surface optimization experiment results

The response surface experimental design was utilized to optimize the extraction process. The analysis results could be found in Table 2 and Table 3. The response surfaces were plotted based on the values of the Os extraction rate (Y), which showed the three experimental factors (A, B, C) obtained after regression fitting had an impact on Os. The regression equation was as follows:

**Table 2:** Response surface results of content of Saponins in oat grain.

No.	A	B	C	Actual measured extraction of Os （%）	Predicted extraction of Os （%）
1	0	0	0	0.300 ± 0.026	3.020
2	0	0	0	0.303 ± 0.078	3.020
3	0	1	1	0.279 ± 0.040	2.780
4	1	0	1	0.273 ± 0.054	2.700
5	0	−1	1	0.263 ± 0.040	2.640
6	0	−1	−1	0.269 ± 0.040	2.710
7	−1	1	0	0.239 ± 0.006	2.380
8	0	0	0	0.300 ± 0.016	3.020
9	−1	0	1	0.245 ± 0.026	2.480
10	−1	−1	0	0.229 ± 0.026	2.250
11	0	0	0	0.297 ± 0.026	3.020
12	0	0	0	0.304 ± 0.036	3.020
13	1	0	−1	0.279 ± 0.039	2.760
14	0	1	−1	0.283 ± 0.031	2.820
15	1	−1	0	0.247 ± 0.015	2.480
16	1	1	0	0.256 ± 0.038	2.600
17	−1	0	−1	0.250 ± 0.039	2.530

**Table 3:** Analysis of variance of regression model equations.

SV	SS	df	MS	*F* value	*P* value
Model	0.970	9	0.110	50.82	<0.0001
A	0.110	1	0.110	49.86	0.0002
B	0.030	1	0.030	14.14	0.0071
C	5.512E-003	1	5.512E-003	2.600	0.1511
AB	2.500E-005	1	2.500E-005	0.012	0.9166
AC	2.500E-005	1	2.500E-005	0.012	0.9166
BC	1.000E-004	1	1.000E-004	0.047	0.8343
A^2^	0.530	1	0.530	248.640	<0.0001
B^2^	0.240	1	0.240	110.970	<0.0001
C^2^	0.0091	1	9.104E-003	4.290	0.0771
Residual	0.015	7	2.122E-003		
Lack of Fit	7.575E-003	3	2.525E-003	1.390	0.3682
Pure Error	7.280E-003	4	1.820E-003		
Cor Total	0.990	16			
R^2^				0.985	
R^2^（Adj）				0.966	
C.V.%				1.690	

Y(%) = 3.03 + 0.12A+0.061B-0.026C-0.0025AB-0.0025AC+0.005BC-0.35A^2^-0.24B^2^-0.047C^2^.

The analysis of variance (Table 3) revealed that the regression model was significant (*P*<0.0001), with an F-value of 50.82. This indicated that the relationship between the independent variables and the response value was highly significant. The lack of fit term represented the degree of difference between the model used and the test. In this case, the *P*-value of 0.368 (less than 0.05) was beneficial to the model, suggesting a good fit and no lack of fit factor. Therefore, the regression equation accurately reflected the actual situation.

The regression equation could be used to analyze the test results. The coefficient of determination (R^2^) for the model was 0.9849, indicating that the model could explain 98.49 % of the changes in the response value. The adjusted determination coefficient (R^2^ Adj) was 0.9655, and the coefficient of variation was 1.69 %. It suggested that 3.45 % of the variation in the model could not be explained by the model. Overall, the model fitted well and could be utilized to analyze and predict the extraction rate of Os.

The *F* value reflected the importance of each factor's influence on the test values. The one-order term coefficient of factor A and B had values of 0.12 and 0.061, respectively, both of which were positive values. The *P* values of factors A and B were 0.0002 and 0.0071, respectively, both of which were less than 0.05, indicating that the extraction temperature and ultrasonic power had a significant impact on the extraction rate of Os. The binomial coefficients of factors A^2^ and B^2^ had values of 0.35 and 0.24, respectively, both of which were negative values. The *P* values of A^2^ and B^2^ less than 0.0001, indicated that A^2^ and B^2^ significantly impacted on the extraction rate of Os. On the other hand, the *P* values of factors C (0.1511) and C^2^ (0.0771) were both greater than 0.05, suggesting that factor C, the material-solvent ratio, did not have a significant impact on the extraction rate of Os.

**Fig. 2:**
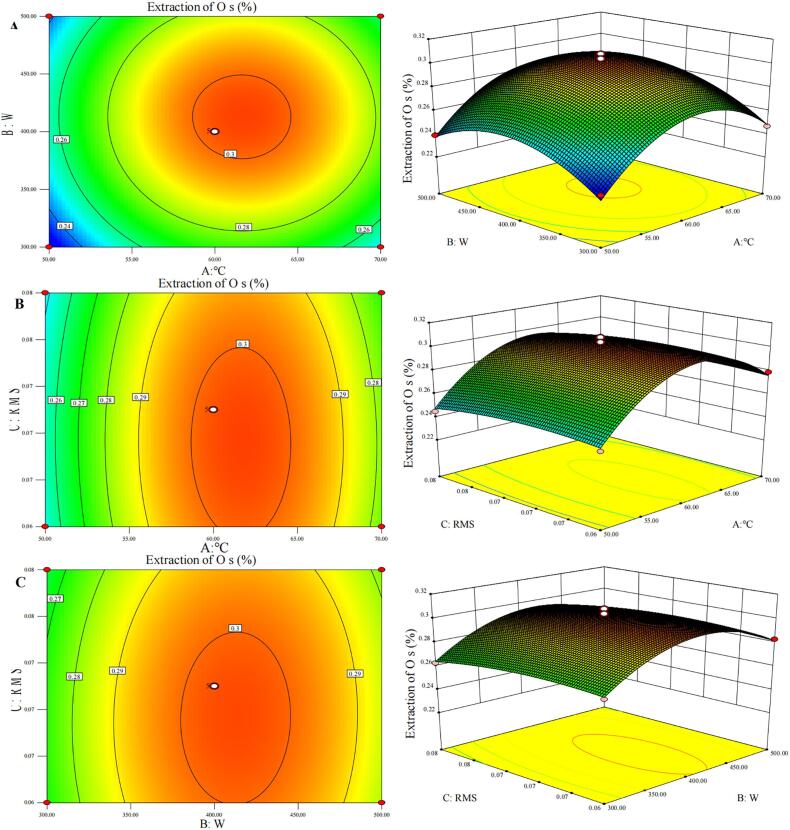
Response surface optimization process contour and response surface 3D map. A.The contours and response surface 3D map of interaction between extraction temperature and ultrasonic time; B. The contour and response surface 3D map of interaction between extraction temperature and solid–liquid ratio; C. The contour and response surface 3D map of interaction between ultrasonic time and solid–liquid ratio. In the figure, RMS represents the ratio of solid to liquid.

To further investigate the interactive effects of extraction time, ultrasonic power, and material-solvent ratio on the saponins extraction rate of oat grains, contour plots and 3D response surface plots were analyzed (Fig. 2). The response surface method provided an intuitive reflection of the influence of two factors on the response value. In the 3D model, a steeper slope indicated a greater impact of the factor on the response value, while a smoother model suggested a smaller impact. In the contour plot, a circular shape indicated a non-significant interaction between the two factors, whereas an elliptical shape suggested a significant interaction.

The results showed in Fig. 2A indicated that the extraction rate of Os initially increased and then decreased with the increase of ultrasonic power when the material-solvent ratio of the fixed system was 1:14. Similarly, as the extraction temperature gradually increased, the extraction rate of Os exhibited a trend of initially increasing and then decreasing. The response surface diagram revealed that both extraction temperature and ultrasonic power had a significant impact on the Os extraction rate, as indicated by the relatively steep surface changes. However, the contour map suggested that the interaction between these two factors had no significant impact on the extraction rate, as depicted by the circular pattern of the contour lines.

In Fig. 2B, when the ultrasonic power of the fixed system was 400 W, the extraction rate of Os initially increased and then decreased as the extraction temperature continued to increase. The maximum extraction rate of Os was observed at approximately 60 °C. Similarly, as the material-solvent ratio increasing, the extraction rate of saponins initially increased and then decreased, although the change was not significant. The response surface diagram revealed that the extraction temperature had a more pronounced effect on the Os extraction rate compared to the material-solvent ratio.

Fig. 2C demonstrates that, when the extraction temperature was set at 60 °C in a fixed system, the extraction rate of Os initially increased and then decreased as the ultrasonic power increased. Meanwhile, the extraction rate of Os did not show a significant change with the increase in material-solvent ratio. The response surface diagram revealed that the ultrasonic power surface changed more steeply compared to the material-solvent ratio surface, indicating that ultrasonic time had a greater impact on the Os extraction rate. Although the contour plots in Fig. 2B and 2C displayed nearly elliptical patterns, the results of the response surface variance analysis suggested that the interaction between the material-solvent ratio and extraction temperature, as well as the material-solvent ratio and ultrasonic time, had a significant impact on the extraction rate of Os, while the rate effect was not particularly significant.

Based on these findings, the optimal extraction conditions for Os were 1:14 of material-solvent ratio, 412.49 W of ultrasonic power, and 61.62 °C of extraction temperature to acquire 0.304 %±0.306 % of the maximum extraction rate of Os.

### 3.2 Evaluation of antioxidant activity for the crude extract of oat saponins

#### 3.2.1 The scavenging or inhibiting abilities of oat saponins to free radicals in vitro

**Fig. 3:**
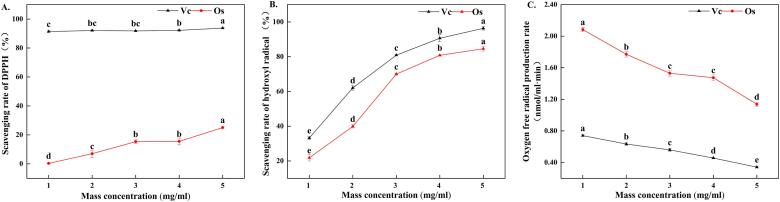
Antioxidant tests of Saponins of oat grain（Os）with different concentrations. A. The effect of different concentrations of Os on scavenging rate of DPPH; B. The effect of different concentrations of Os on scavenging rate of hydroxyl free radical; C. The effect of different concentrations of Os on the oxygen free radical production rate.

The crude Os extract had substancial scavenging or depressing effects on three free radicals (1,1 Dipheny1 2 picry1 hydrazy1 (DPPH); hydroxyl radical (·OH); superoxide anions (O_2_^–^·), as shown in Fig. 3A, 3B and 3C. The antioxidant abilites of the crude Os extract increased with the concentration of Os. The highest scavenging or inhibiting rate of Os to DPPH, ·OH and O_2_^–^· free radicals could reach up to 25.06 %, 84.59 % and 0.34 nmol/mL·min, respectively, but were lower than that of Vc (93.80 %, 96.33 % and 1.14 nmol/ml·min, respectively). Interestingly, the scavenging rate of Os to ·OH free radical was very closer to that of Vc.

#### 3.2.2 Anti-protein oxidation capacity of oat saponins during pork storage

##### 3.2.2.1 The changes of protein content

**Fig. 4:**
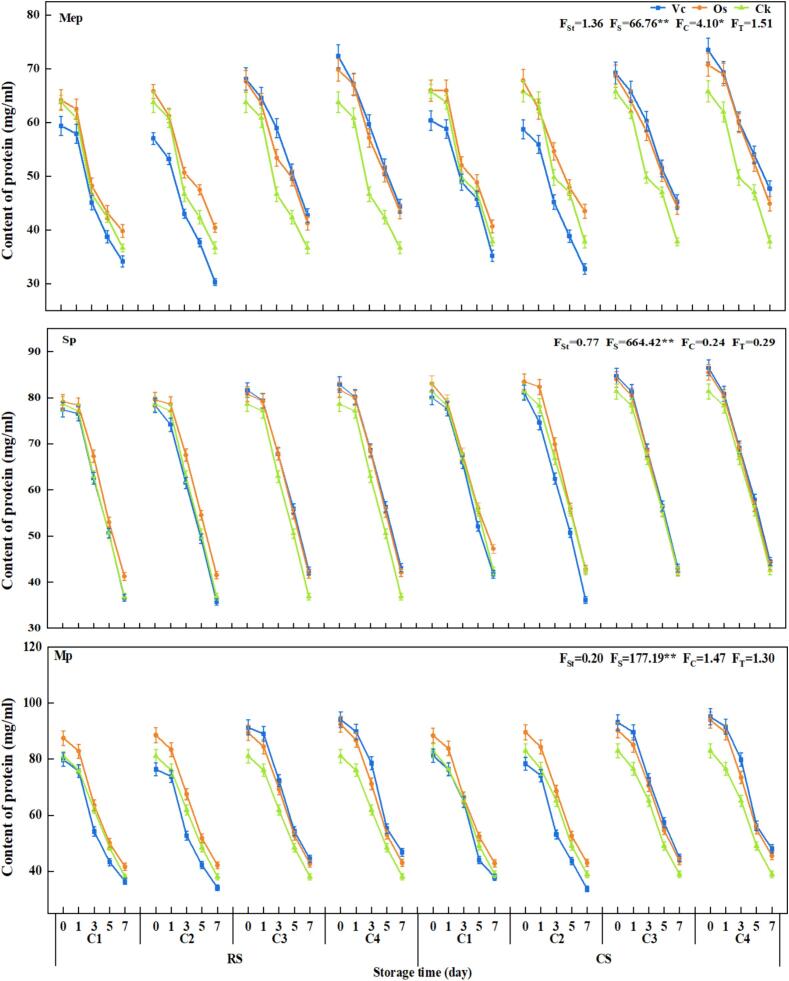
Effects of different treatments on the content of protein. Note: *: indicates a significant difference at *P*<0.05; **: indicates a highly significant difference at *P*<0.01. Muscle proteins = Mep; Sarcoplasmic proteins = Sp; Myofibrillar proteins = Mp; Room Storage (23℃) = RS; Cool Storage (4℃) = CS; F_St_ = The ANOVA F value of different storage temperature; F_S_=The ANOVA F value of different storage days; F_C_=The ANOVA F value of different concentration of treatments; F_T_=The ANOVA F value of different treatments; L-ascorbic acid = Vc, Oat-saponins = Os, Add sterile water = Ck; C1 = 0.09 mg/mL; C2 = 0.18 mg/mL; C3 = 0.36 mg/mL; C4 = 0.72 mg/mL. The same below.

In order to further clarify the inhibitory effect of Os on protein oxidation, a pork storage experiment was conducted. The results indicated that the Mep, Sp and Mp contents of all groups showed a significant decreasing trend with the storage time under the conditions of room temperature or cool storage (Fig. 4). The third day of storage marked the onset of intense oxidation, resulting in a significant decrease in protein content compared with the initial state. Specifically, Mp decreased by 16.94 % to 32.12 %, Mep and Sp decreased by 11.48 % to 26.85 % and 11.30 % to 20.51 %, respectively. On the seventh day of storage, intense oxidation of the second stage occurred, leading to the maximum protein degradation and reaching the lowest protein content across all samples (32.74–48.09 mg/mL). Notably, there was a relatively substantial degradation observed for Sp and Mp at approximately 44.30 % −53.89 % and 40.96 % −58.00 %, respectively. In contrary, Mep exhibited a smaller range of degradation between 20.98 % −49.01 %.

During the same storage period, when the treatment concentration was below C2 (0.36 mg/ml), the protein content in each group treated with Os was higher, resulting in the most effective antioxidant effect. Conversely, when the treatment concentration exceeded C2 (0.36 mg/ml), the Vc group exhibited a significant inhibitory effect on protein oxidation. After 3 days of storage, the contents of Mep, Sp and Mp in the CK group were lower than those in both Vc and Os groups. This indicated that high-concentration Vc and Os treatments could effectively inhibit pork protein oxidation, with a stronger inhibition observed as saponins concentration increasing.

Among the treatments, the protein contents in the CK group exhibited the lowest levels (50.06–––64.88 mg/mL), while protein degradation in both Vc and Os treatment groups showed varying degrees of alleviation, reduced by 7.76 % to 27.98 % in the Vc treatment group and by 7.82 % to 22.53 % in the Os treatment group. Within the experimental concentration range, high-concentration treatments within each group demonstrated superior efficacy in mitigating protein degradation.

##### 3.2.2.2 The changes of sulfhydryl content

**Fig. 5:**
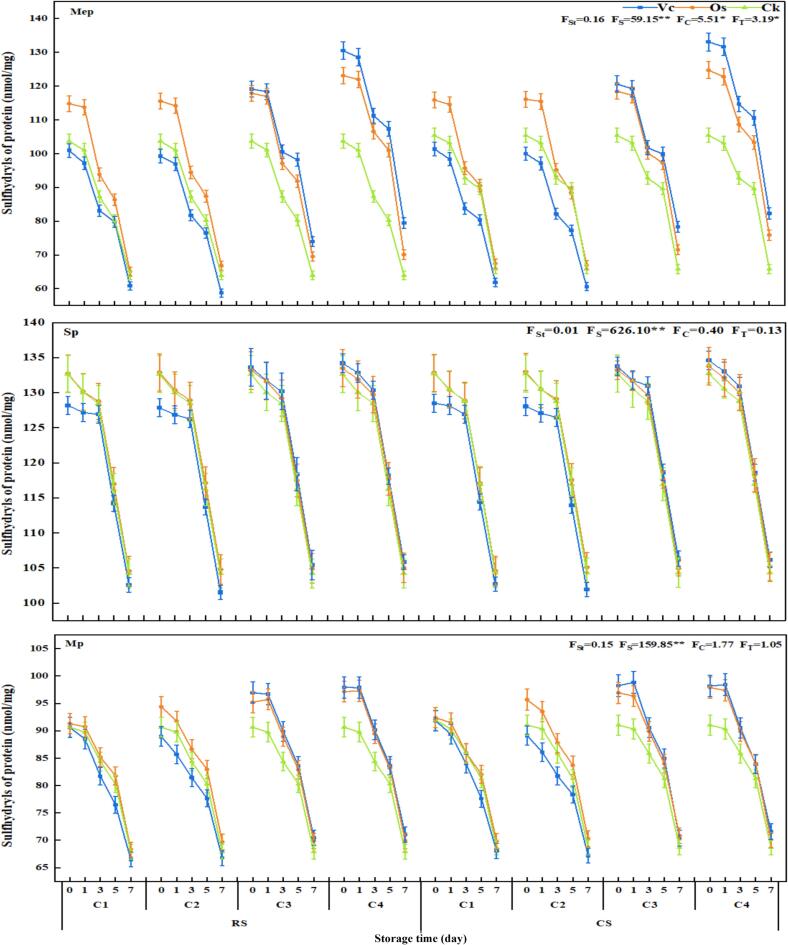
Effects of different treatments on the total sulfhydryl group content.

The sulfhydryl is a main functional group in protein structure. The total sulfhydryl content of all groups decreased gradually with the extension of storage time, and at the initial stage of storage, all groups exhibited a high level of sulfhydryl group content. The sulfhydryl contents of Mep, Sp, and Mp ranged from 100.90 to 133.09, 128.20 to 133.77, and 90.62 to 98.16 nmol/mg, respectively (Fig. 5). The first oxidation peak occurred on day 3 of storage when the total sulfhydryl contents of Mep, Sp and Mp decreased in the range of 81.74–114.67 nmol/mg, 126.26–130.92 nmol/mg, and 81.44–90.51 nmol/mg, respectively. It could be observed that compared with Sp and Mep, Mp significantly reduced the total sulfhydryl content while its oxidation rate was significantly accelerated. On day 7 of storage, the sulfhydryl group content reached its lowest point in each group (ranging from 63.94 to104.22 nmol/mg), with Mp showing the largest reduction (17 % −28 %).

The oxidation degree of different protein groups varied with treatment concentrations. When the treatment concentration was below C2, the Os groups exhibited superior antioxidant capacity. As the treatment concentration increased, the antioxidant capacity gradually intensified. When the treatment concentration exceeded C2, the Vc groups demonstrated the strongest antioxidant capacity, and high concentrations of Vc and saponins could effectively reduce protein oxidation to varying extents. Among all groups, only Mep showed a significant difference in treatment concentration regarding the total sulfhydryl group content.

Among the treatments, CK groups exhibited the highest degree of oxidation and the lowest sulfhydryl content (58.73–101.93 nmol/mg). Both the Vc and Os treatment groups demonstrated varying degrees of alleviation in protein degradation, reducing protein degradation by 1.17 %–33.78 % and by 1.29 %-25.86 %, respectively. Furthermore, within the concentration range specified by the test, high-concentration treatments from each respective group yielded optimal reduction in protein degradation.

##### 3.2.2.3 The changes of carbonyl concent

**Fig. 6:**
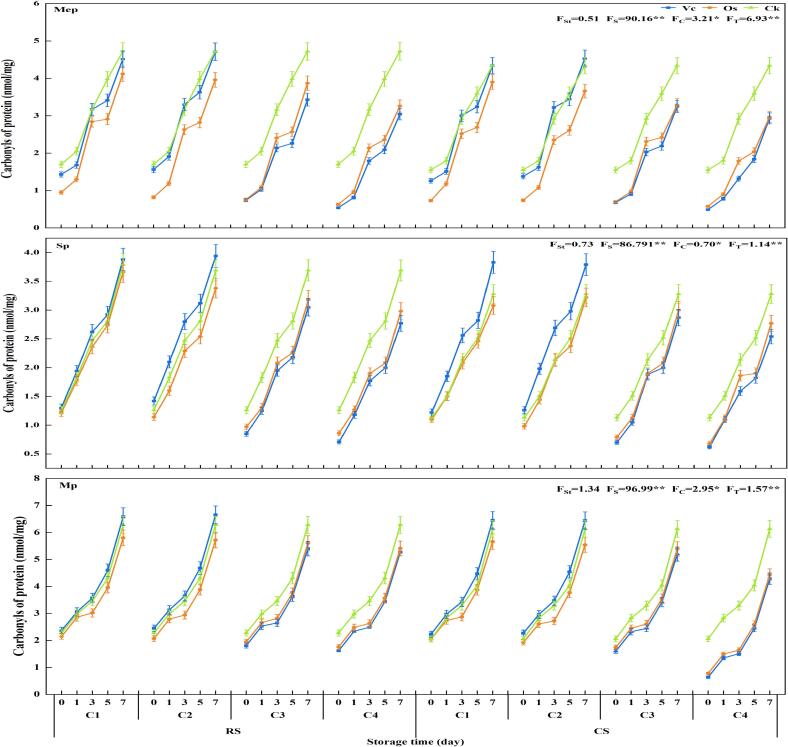
Effects of different treatments on the carbonyl concent.

The carbonyl is a product in protein oxidation process. The carbonyl contents significantly increased at different storage temperatures as the storage time extended (Fig. 6). The carbonyl content in all groups was below 2 nmol/mg during the initial storage stage. On the 3rd day of storage, a significant increase in carbonyl content was observed, with Mep, Sp and Mp increasing by 86.63 %-242.90 %, 88.04 %-172.56 % and 39.67 % −312.10 %, respectively. On the 7th day of storage, each group reached its maximum carbonyl content (4.71–6.65 nmol/mg), with Mp showing the highest increase (169.94 % to 571.56 %), followed by Mep and Sp (178.51 % to 497.05 % and 177.36 % to 312.48 %, respectively).

The differences among different treatment concentrations in all groups reached a significant level (*P* < 0.05). Within the experimental concentration range, there was a significant decrease in carbonyl content with increasing concentration. When Vc concentration was lower than C2, the Vc treatment group exhibited the highest carbonyl content while the Os treatment group showed the lowest. Conversely, when Vc concentration was higher than C2, the carbonyl content in each group of CK exceeded that of the other two groups significantly, indicating that the degree of protein oxidation in CK group was the most severe and that the addition of Vc and Os for pork had certain antioxidant effects.

Among the treatments, CK group exhibited the highest carbonyl content (3.28–6.28 nmol/mg). Both the Vc and Os treatment groups demonstrated varying degrees of alleviation in protein degradation and reduction in carbonyl content. Specifically, the Vc treatment group showed a decrease in carbonyl groups ranging from 12.26 % to 54.62 %, while the Os treatment group exhibited a reduction ranging from 8.49 % to 50.05 %. Within the concentration range tested, high-concentration treatments within each group yielded superior effects on reducing carbonyl content.

#### 3.2.3 Anti-protein oxidation capacity of oat saponins in Fenton oxidation system

In order to further understand the effect of hydroxyl radical on pork Mp, the Fenton oxidation system was tested. The results showed that the content of Mp total sulfhydryl group was obviously different under different H_2_O_2_ concentration (Table 4). In the concentration range of 0–5 mM, with the increase of H_2_O_2_ concentration, the oxidation was intensified, and the sulfhydryl content decreased. At the concentration of 1 mM, the sulfhydryl content was significantly different from that of the first five concentrations, decreasing to 13.09 nmol/mg and reaching the lowest level; at the concentration of 5 mM, the sulfhydryl content reached the lowest level (8.08 nmol/mg). Proper rinsing could reduce protein oxidation, and the total sulfhydryl group content in all rinsing groups was significantly higher than that in non-rinsing groups. The results showed that the hydroxyl radical oxidation system could oxidize pork Mp.

**Table 4:** Effects of different treatments on the contents of total sulfhydryl and carbonyls in the oxidation of Mp induced by Fenton oxidation system.

Code	Treatments	Total sulfhydryl content	Carbonyls content
1	Only the Mp solution(CK)	58.4 ± 0.17c	5.68 ± 0.35a
2	Add Os = 0.72 mg/mL in the Mp solution	77 ± 0.07a	2.83 ± 0.49c
3	Add Vc = 0.72 mg/mL in the Mp solution	86.46 ± 0.28a	2.60 ± 0.21c
4	only the Mp solution under Fenton oxidation systems	35.88 ± 0.06e	6.86 ± 0.02b
5	Add Os = 0.09 mg/mL in the Mp solution under Fenton oxidation systems	48.36 ± 0.11d	4.71 ± 0.07bc
6	Add Os = 0.18 mg/mL in the Mp solution under Fenton oxidation systems	51.92 ± 0.17c	2.96 ± 0.11c
7	Add Os = 0.36 mg/mL in the Mp solution under Fenton oxidation systems	64.92 ± 0.02b	1.69 ± 0.1d
8	Add Os = 0.72 mg/mL in the Mp solution under Fenton oxidation systems	78.72 ± 0.12a	0.93 ± 0.04e

Different letters in a column indicate significant differences (ANOVA, *P*<0.05) among different treatments.

In order to further understand the scavenging ability of Vc and Os on hydroxyl radical, the effect of Fenton oxidation system on protein oxidation during the strong oxidation of pork Mp was studied. The results showed that Os had a certain inhibitory effect on Mp oxidation, and the higher the concentration, the stronger the inhibitory effect. In the treatment without enhanced oxidation system (Code 1), adding only Os (Code 2) and Vc (Code 3) could inhibit Mp oxidation to different degrees. The results of total sulfhydryl and carbonyl groups also supported this conclusion.

## 4 Discussion

### 4.1 Optimization of ultrasonic assisted extraction of oat saponins

Ultrasonic-assisted extraction has the advantages of short operation time, low solvent consumption, high yield of active components, and low environmental pollution and so on. It has been widely used in the extraction of active components of natural products [32]. Ethanol concentration, solid–liquid ratio, ultrasonic time, ultrasonic power, extraction time and extraction temperature had significant effects on the extraction rate of saponins in the single factor test. As an organic solvent, ethanol facilitates the dissolution of saponins [33]. Thus, increasing the concentration of ethanol may be helpful for the dissolution and extraction of saponins. However, it should be noted that too high concentration of ethanol may cause the degradation or precipitation of saponins, which will reduce the extraction efficiency. Therefore, it is necessary to select suitable ethanol concentration [34].

In addition to ethanol concentration, solid–liquid ratio is also an important factor affecting the extraction efficiency. Too low solid–liquid ratio may result in insufficient contact between saponins and ethanol, limiting the extraction efficiency [35]. Therefore, it is necessary to select an appropriate solid–liquid ratio according to the specific situation to balance the relationship between extraction efficiency and solvent utilization when extracting [17]. The adjustment of ultrasonic time and power has an impact on the thermal and cavitation effects of ultrasonic waves in the extraction process, which can promote the release and dissolution of saponins, thus improving the extraction efficiency [36], [37], [38], [39]. However, it should be noted that too high ultrasonic power or too long ultrasonic time may lead to a decline in the quality of the active components in the extract or the degradation of some components [40], so proper control and regulation is required. In addition, longer extraction time and higher temperature are generally beneficial to increase the extraction rate. It is because that longer extraction time and appropriate temperature help the solvent to fully contact and react with the target component in the raw material [41]. However, too high temperature may accelerate the degradation of saponins, reducing the extraction efficiency [42]. Therefore, the extraction rate and the stability of raw materials should be considered comprehensively, and appropriate conditions should be selected for extraction.

### 4.2 Anti-protein oxidant properties of oat saponins

The results of in vitro antioxidant experiments showed that the scavenging effect of oat saponins on DPPH, hydroxyl radical and superoxide anion was not as good as that of Vc, but the scavenging effect on hydroxyl radical was the closest to that of Vc. The predominant saponins found in oat seeds are steroid saponins, which consist of a hydrophobic aglycone skeleton and a hydrophilic glycosyl side chain. The glycosyl side chain serves as the primary functional group responsible for its antioxidant activity, and its structure exhibits relative stability. Vc refers to ascorbic acid, which contains a dienol (hydroxyl) moiety within its structure that is highly labile in aqueous solutions and prone to oxidation, particularly at low concentrations [43].

Based on the above results, the crude extract of oat saponins was applied in pork storage and Fenton reaction system, because pork is more vulnerable to hydroxyl radical attack and leads to protein oxidation during storage, and Fenton reaction system is a strong protein oxidation system. The results showed that Os could inhibit the protein oxidation caused by hydroxyl radical and the inhibition increased with Os concentration., But the inhibition ability of the control Vc to protein oxidation showed the contrary under the lower or higher concentrations. The reason may be that Os and Vc have different chemical structures, which may cause them to react with different mechanisms and efficiencies with free radicals [44].

The interaction between free radicals and proteins in meat can result in the modification of protein peptide chains, amino acid side chains, and covalent cross-linking among protein molecules, leading to protein oxidation. Among various free radical inducers, hydroxyl radicals are the most prevalent agents responsible for inducing protein oxidation. Vc is a powerful antioxidant, and its molecular structure contains multiple hydroxyl (·OH) groups, which can react with free radicals to form stable products, thereby removing free radicals [45]. As an antioxidant addition, its removal effect can only play a role in the concentration greater than 2 mM. Otherwise, it will promote the REDOX cycle between metal ions and proteins. More hydroxyl radicals are produced and oxidation will be accelerated [43], which is consistent with our results. The mechanism of Os to inhibit protein oxidation may be to interrupt the attack of free radicals on aliphatic amino acid side chain groups [46]. It may be because Os reduces lipid oxidation to a certain extent and the substrate that indirectly induces protein oxidation [47]. It may also be because a series of microorganisms are produced during meat storage, and the enzymes secreted by these microorganisms decompose Os, which may occupy the site of sulfhydryl reaction in the process of decomposition and synthesis of these substances. It avoids the cross-linked polymerization of proteins due to oxidation. In addition, the antioxidant activity of saponins may also be affected by other factors, including reaction rate and solubility [48]. Os may show different antioxidant effects from Vc affected by the combination of these factors when reacting with free radicals. However, the specific response mechanism needs to be further studied.

## 5 Conclutions

To sum up, ultrasonic-assisted extraction method proved to be highly effective in extracting Os from oat seed. The optimal extraction conditions were ethanol volume fraction of 80 %, material-solvent ratio of 1:14, ultrasonic power of 400 W, ultrasonic time of 25 min, extraction temperature of 60℃, extraction time of 180 min. In addition, this work found that the DPPH, ·OH and O^2–^ free radical activities reduced with increasing concentration of the Os. Furthermore, the antioxidant capacity of Os in pork storage and Fenton oxidation system was revealed, respectively. The results showed that Os could effectively inhibit the oxidation of protein. In this work, the extraction technology and a multidimensional evaluation of the antioxidant capacity of oat seed saponins were analyzed to contribute to the selection of modern processing technologies for realizing the industrialization and application of saponins. This extraction technology also offers a new-sustainable solution for repurposing oat by-products (bran, stalk) and aligns with the principles of a circular bioeconomy.

## CRediT authorship contribution statement

**Lina Zhang:** Conceptualization, Data curation, Formal analysis, Investigation, Methodology, Resources, Visualization, Writing – original draft, Writing – review & editing. **Jianing Li:** Formal analysis, Investigation, Visualization. **Yingrui Huo:** Investigation, Resources. **Wenping Yang:** Writing – review & editing. **Jie Chen:** Supervision, Validation. **Zhiqiang Gao:** Funding acquisition, Project administration, Supervision. **Zhenping Yang:** Conceptualization, Data curation, Formal analysis, Funding acquisition, Methodology, Project administration, Supervision, Validation, Writing – review & editing.

## Declaration of competing interest

The authors declare that they have no known competing financial interests or personal relationships that could have appeared to influence the work reported in this paper.
