# Supplementary material for: Ultrasonic extraction and antioxidant evaluation of oat saponins
Source: Ultrason Sonochem. 2024 Jul 18;109:106989. doi: 10.1016/j.ultsonch.2024.106989 (PMC11327440; doi:10.1016/j.ultsonch.2024.106989)
Supplement: Supplementary Data 1 [file mmc1.doc]

Table 1 Single-factor experimental design


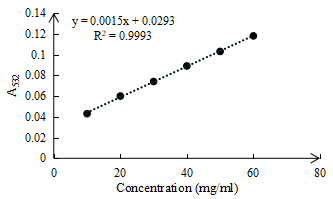


Figure 1. Standard curve

| Factor | Concentration of ethanol  （%） | Ratio of material and solvent | Extracting time by ultrasonic  （min） | Ultrasonic power  （W） | Extracting time  （min） | Extracting temperature  （℃） |
| --- | --- | --- | --- | --- | --- | --- |
| 1 | 40,50,60,  70,80,90 | 1:12 | 20 | 400 | 180 | 60 |
| 2 | 80 | 1:8,1:10,  1:12,1:14,  1:16,1:18 | 20 | 400 | 180 | 60 |
| 3 | 80 | 1:12 | 5,10,15,  20,25,30 | 400 | 180 | 60 |
| 4 | 80 | 1:12 | 20 | 240,300,348,  400,500,600 | 180 | 60 |
| 5 | 80 | 1:12 | 20 | 400 | 60,90,120  150,180,210 | 60 |
| 6 | 80 | 1:12 | 20 | 400 | 180 min | 30,40,50,  60,70,80, |

Table 2 Orthogonal experimental design

| Factor | 1 Level | 0 Level | -1 Level |
| --- | --- | --- | --- |
| A （Extracting temperature） | 50 | 60 | 70 |
| B（Ratio of material and solvent） | 1:12 | 1:14 | 1:16 |
| C（Ultrasonic power） | 300 | 400 | 500 |

Table 3 Test factors and levels of response surface for extraction of Os

| Factors | Level | | |
| --- | --- | --- | --- |
|  | -1 | 0 | 1 |
| A. Extracting temperature/℃ | 50 | 60 | 70 |
| B. Ultrasonic power/W | 300 | 400 | 500 |
| C. Ratio of material and solvent | 1:12 | 1:14 | 1:16 |

Table 4 Orthogonal test results (three-factor, three-level orthogonal regression)

| No. | A | B | C | Extraction of Os（%） | No. | A | B | C | Extraction of Os（%） |
| --- | --- | --- | --- | --- | --- | --- | --- | --- | --- |
| 1 | 1 | 1 | 1 | 0.241±0.040 | 19 | -1 | 1 | 1 | 0.223±0.026 |
| 2 | 1 | 1 | 0 | 0.256±0.040 | 20 | -1 | 1 | 0 | 0.239±0.010 |
| 3 | 1 | 1 | -1 | 0.224±0.026 | 21 | -1 | 1 | -1 | 0.216±0.015 |
| 4 | 1 | 0 | 1 | 0.279±0.040 | 22 | -1 | 0 | 1 | 0.250±0.040 |
| 5 | 1 | 0 | 0 | 0.289±0.040 | 23 | -1 | 0 | 0 | 0.281±0.026 |
| 6 | 1 | 0 | -1 | 0.273±0.054 | 24 | -1 | 0 | -1 | 0.245±0.026 |
| 7 | 1 | -1 | 1 | 0.229±0.026 | 25 | -1 | -1 | 1 | 0.216±0.015 |
| 8 | 1 | -1 | 0 | 0.247±0.015 | 26 | -1 | -1 | 0 | 0.229±0.026 |
| 9 | 1 | -1 | -1 | 0.218±0.178 | 27 | -1 | -1 | -1 | 0.205±0.040 |
| 10 | 0 | 1 | 1 | 0.275±0.030 | 28 | -1.215 | 0 | 0 | 0.228±0.030 |
| 11 | 0 | 1 | 0 | 0.287±0.026 | 29 | +1.215 | 0 | 0 | 0.238±0.174 |
| 12 | 0 | 1 | -1 | 0.264±0.040 | 30 | 0 | -1.215 | 0 | 0.218±0.129 |
| 13 | 0 | 0 | 1 | 0.285±0.015 | 31 | 0 | +1.215 | 0 | 0.279±0.040 |
| 14 | 0 | 0 | 0 | 0.300±0.026 | 32 | 0 | 0 | -1.215 | 0.231±0.054 |
| 15 | 0 | 0 | -1 | 0.276±0.015 | 33 | 0 | 0 | +1.215 | 0.248±0.030 |
| 16 | 0 | -1 | 1 | 0.269±0.040 | 34 | 0 | 0 | 0 | 0.297±0.026 |
| 17 | 0 | -1 | 0 | 0.279±0.015 | 35 | 0 | 0 | 0 | 0.303±0.028 |
| 18 | 0 | -1 | -1 | 0.263±0.040 |  |  |  |  |  |
